# The Status of Hospital Domestic Service

**Published:** 1920-08-21

**Authors:** 


*To the Editor of* The Hospital.

### The Status of Hospital Domestic Service.

*To the Editor of* The Hospital.

Sir,—In this week's issue of The Hospital your correspondent, Gladys M. E. Leigh, says, "It is as futile to suggest that domestic service in a hospital should be a stepping-stone into the nursing service as it would be to suggest that a R.A.M.C. orderly might advantageously qualify in medicine." I should like to say that I fail to see why a R.A.M.C. orderly, given the necessary funds and environment, and naturally endowed with the brains and that sense of honour and chivalry so necessary to the profession, should *not* qualify in medicine; just as I am at a loss to understand why the naturally refined and clever daughter of a manual worker should not make an excellent sick nurse if she feels that to be her vocation and undergoes the necessary training. One might as well say it were futile to suggest that a once humble grocer is to-day Sir Thomas Lipton, or that the orphaned nephew of a shoemaker is the present Prime Minister of England.—Yours faithfully,

August 16, 1920. "A Worker."

